# A Study on the Effects of Selenization Temperature on the Properties of Na-Doped Cu_2_ZnSn(S,Se)_4_ Thin Film and Its Correlation with the Performance of Solar Cells

**DOI:** 10.3390/nano11092434

**Published:** 2021-09-18

**Authors:** Zhanwu Wang, Dongyue Jiang, Fancong Zeng, Yingrui Sui

**Affiliations:** 1 Department of Life Sciences, Jilin Normal University, Siping 136000, China; wangzhanwu@126.com; 2 Department of Physics, Jilin Normal University, Siping 136000, China; Jiangdongyue9857@163.com (D.J.); ZENG740183899@163.com (F.Z.)

**Keywords:** Cu_2_ZnSn(S,Se)_4_ film, Na doping, selenization temperature, properties, solar cells

## Abstract

In this study, we prepared Na-doped Cu_2_ZnSn(S,Se)_4_ [noted as (Na_0.1_Cu_0.9_)_2_ZnSn(S,Se)_4_] films on the Mo substrate using a simple and cheap sol–gel method together with the post-annealing technique. The effects of selenization temperature on the properties of Na-doped Cu_2_ZnSn(S,Se)_4_ were surveyed. The results indicated that some sulfur atoms in the films were substituted by selenium atoms by increasing the selenization temperature, and all films selenized at different temperatures had a kesterite structure. As the selenization temperature increased from 520 to 560 °C, the band gaps of the film can be tuned from 1.03 to 1 eV. The film with better morphology and opto-electrical properties can be obtained at an intermediate selenization temperature range (e.g., 540 °C), which had the lowest resistivity of 47.7 Ω cm, Hall mobility of 4.63 × 10^−1^ cm^2^/Vs, and carrier concentration of 2.93 × 10^17^ cm^−3^. Finally, the best power conversion efficiency (PCE) of 4.82% was achieved with an open circuit voltage (Voc) of 338 mV, a short circuit current density (Jsc) of 27.16 mA/cm^2^ and a fill factor (FF) of 52.59% when the selenization temperature was 540 °C.

## 1. Introduction

Cu_2_ZnSn(S,Se)_4_ (CZTSSe) is a helpful absorber layer for high reserves and inexpensive thin film solar cells because of its appropriate semiconductor characteristics having a tunable band gap, p-type conduction characteristics, and high absorption coefficient [1,2,3]. Furthermore, it can be considered as a substitute to Cu(In, Ga)Se_2_ and CuInS_2_ absorbers, in which the extremely rare and expensive indium (In) is substituted by abundant and environmentally friendly zinc (Zn) and tin (Sn) [4,5,6]. At present, studies on Cu_2_ZnSnS_4_ (CZTS)-based solar cells are still in their infancy, with the highest reported PCE of 12.62% [7], which is far below the theoretical PCE of 32.2% [8], and at the same time, it also is far behind the PCE (22.9%) of CuInGaSe_2_ (CIGSe) counterparts [9]. The PCE of CZTS-based solar cells has not been greatly improved in recent years. There are several factors impeding the efficiency of further improvement for CZTSSe solar cells, including the crystal quality and optoelectronic properties of the CZTSSe semiconductors, as well as the energy band matching between the CZTSSe and CdS interface [10,11,12,13]. Recent research has shown that the fabrication of adjustable band gap absorbers is a practical way to solve the band matching of CdS and CZTSSe heterojunction interfaces and improve cell efficiency. Therefore, many studies on cationic replacement have been performed to adjust the band gap of the CZTSSe, for instance, Ge_Sn_ and Ag_Cu_ [14,15,16]. Although Ge_Sn_ and Ag_Cu_ can modulate the band gap of the CZTS, they are too expensive to be used as an ideal absorbing layer material for future commercial production. It was reported that the band gap of the CZTSSe was successfully adjusted by Na doping. In addition, Na doping can significantly improve the crystallization quality and optical–electrical characteristic of CZTSSe [17,18]. These similar results about Na doping have been observed in our previous work. Compared to Ge and Ag, the Na is cheaper, plentiful, and environmentally friendly. Thus, it is deduced that Na-doped Cu_2_ZnSn(S,Se)_4_ is more worthy of studying as a potential absorbing layer material.

It is well known that in order to obtain CZTSSe films with a single phase and uniform dense grains, the precursor film prepared by spin coating is usually heat-treated at a higher temperature. This indicated that the selenization temperature of the film plays a very important role in enhancing performance, such as crystal quality and photoelectric properties [19,20,21]. The impact of the selenization temperature on the optical-electrical characteristic of CZTSSe has been widely researched [22,23,24]. However, the impacts of the selenization temperature on the structural and photoelectric characteristic of Na-doped Cu_2_ZnSn(S,Se)_4_ films have not been researched to this day. In this work, we have reported the preparation of highly crystalline Na-doped Cu_2_ZnSn(S,Se)_4_ films utilizing a sol–gel spin-coating technique together with the selenization process. In addition, the impact of the selenization temperature on the structure and optoelectronic properties of Na-doped Cu_2_ZnSn(S,Se)_4_ as well as the device performance have been investigated in detail.

## 2. Experimental Details

### 2.1. Preparation of (Na_0.1_Cu_0.9_)_2_ZnSn(S,Se)_4_ Film

For the preparation of Na-doped Cu_2_ZnSn(S,Se)_4_ films, the precursor solution was obtained by dissolving copper (II) acetate monohydrate (5.400 × 10^−3^ mol), tin (II) chloride dihydrate (3.750 × 10^−3^ mol), zinc acetate (4.396 × 10^−3^ mol), sodium chloride (0.600 × 10^−3^ mol), thiourea (3 × 10^−2^ mol) and monoethanolamine (MEA) in dimethyl sulfoxide. The precursor solution was incessantly stirred at a temperature of 45 °C to make the metal compounds completely dissolve. Thereafter, the solution was spun onto the Molybdenum-coated soda lime glasses (SLGs) at a speed of 3000 rpm for 30 s, then the substrate was dried at 300 °C in the air atmosphere. In the end, the Na-doped Cu_2_ZnSn(S,Se)_4_ precursor films were annealed at different temperatures for 15 min in the selenium atmosphere. 

### 2.2. Device Fabrication

The Na-doped Cu_2_ZnSn(S,Se)_4_ thin films were prepared by spin coating a precursor solution onto a Mo-coated Na-free substrate, then the film was annealed at 540 °C for 15 min in an Se atmosphere. The CdS buffer layer was synthesized using a chemical-bath deposition (CBD). The reaction was accomplished in 190 mL of deionized water with 51.3 mg of Cd(SO)_4_, 10.7 mg NH_4_Cl, 10 mL NH_3_·H_2_O, and 76.12 mg thiourea at 75 °C for 14 min. The intrinsic zinc oxide/aluminum zinc oxide (AZO) was deposited as a transparent conducting layer by using a sputtering system. The Ag gate electrode (9 devices per substrate) was then evaporated to complete the device fabrication. The prepared devices were mechanically scribed into nine areas of a 0.19 cm^2^ area.

### 2.3. Characterization

Diffraction patterns of the selenized Na-doped Cu_2_ZnSn(S,Se)_4_ were measured using X-ray power diffraction (XRD, Rigaku Corporation, Tokyo, Japan) and equipped Cu Kα radiation. Raman spectra were surveyed with an excitation wavelength of 514 nm. The chemical component analysis of the films was measured by X-ray photoelectron spectroscopy (XPS; Thermo Fisher Scientific, Waltham, MA, USA) and energy-dispersive X-ray spectroscopy (EDS, JEOL Ltd., Tokyo, Japan). SEM and TEM images were taken using an FEI Nova NanoSEM 230 (Hitachi S-4800, JEOL Ltd., Tokyo, Japan). The electrical properties were performed by a Hall measurement system (Hall, Lakershore HMS 7707, Irvine, CA, USA). The optical spectra were measured using UV–Vis–NIR spectra. The external quantum efficiency (EQE) spectrum was measured using SPIQE200-5324 (QEX10, Newport, Irvine, CA, USA) in air. The current density–voltage characteristics (J–V) of Na-doped Cu_2_ZnSn(S,Se)_4_ solar cells were measured using a 94043A I-V Station under simulated AM 1.5G solar irradiation at a light intensity of 100 mW/cm^2^ (Model 91160, Newport, Irvine, CA, USA).

## 3. Results and Discussion

**Figure 1:**
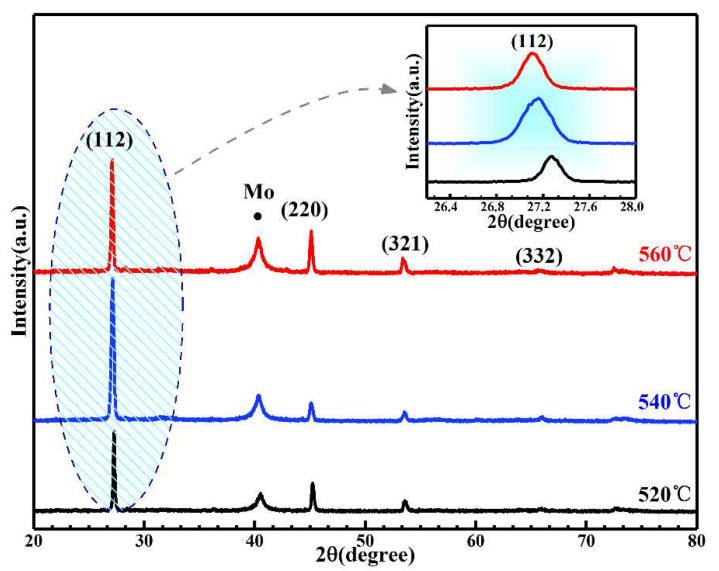
XRD patterns of Na-doped Cu_2_ZnSn(S,Se)_4_ films selenized at different temperatures. Inset: enlarged spectra of (112) diffraction peaks.

Figure 1 displays the XRD patterns of the three (Na_0.1_Cu_0.9_)_2_ZnSn(S,Se)_4_ annealed at the temperatures of 520, 540, and 560 °C (labeled as samples T520, T540, and T560). For all films, five distinct diffraction peaks, located at 27.27°, 45.20°, 53.61°, 66.04°, and 72.70° other than Mo peak (40.53°), can be observed and corresponded to the diffraction peaks of the (112), (220), (312), (008) and (323) lattice planes of the CZTS with kesterite structures [25,26]. Except for the CZTS and Mo diffraction peaks, no other secondary phase was detected, implying that kesterite structures (Na_0.1_Cu_0.9_)_2_ZnSn(S,Se)_4_ were successfully synthesized. In addition, it is observed that the intensity of the (112) peaks for the film selenized at 540 °C is larger than that of other samples, indicating the diffraction peak intensity increases and then decreases with the selenization temperature increasing. This may be because of the increase in the selenization temperature; the grains in the thin films grew stably as they increasingly obtained more energy. Thus, the intensity of the diffraction peak became larger. However, when the selenization temperature increased to a certain value, some atoms had adequate energy to escape from the thin films, which made the defects increase in the thin films. Thus, the peaks’ intensity became weak. We can explore the impact of selenization temperature on the lattice constant by observing the move of the diffraction angle. As shown in the inset of Figure 1, the change in the diffraction angle is linearly dependent on the annealing temperature. All (112) diffraction peaks shifted to small diffraction angles with increased selenization temperatures. Because the decrease in diffraction angles stands for the increase in the lattice distance, it is attributed that plenty of Se occupied the position of S with the temperature increase, which is proven in the EDX results later. These results show that the crystal structure of Na-doped Cu_2_ZnSn(S,Se)_4_ is not changed with the change in the selenization temperature, and when the selenization temperature reached 540 °C, the crystal quality was optimal.

**Figure 2:**
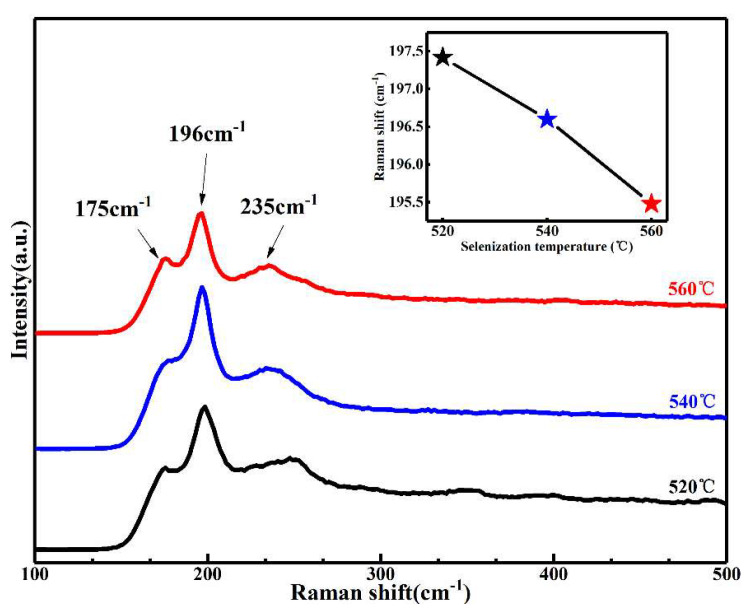
Raman patterns of Na-doped Cu_2_ZnSn(S,Se)_4_ films selenized at different temperatures. Inset: location tracking of the main Raman peak of the A1 mode.

As everyone knows, the detection of the kesterite CZTSSe merely by XRD is not easy, because the XRD feature peaks of the kesterite CZTSSe with some impurity phase chalcogenides are almost overlapped [27]. Therefore, to further determine the purity of the phase and the crystal quality of the Na-doped Cu_2_ZnSn(S,Se)_4_ films, it is usually necessary to characterize the Raman spectrum. Figure 2 depicts the Raman spectroscopy of the Na-doped Cu_2_ZnSn(S,Se)_4_ annealed at different temperatures of 520, 540, and 560 °C (labeled as samples T520, T540, and T560, respectively). The three Raman peaks at 175, 196, and 337 cm^−1^ match the Raman characteristic vibrational A_2_, A_1_, and E modes of the kesterite CZTSSe phase [28,29,30]. No Raman peaks of some possible secondary phases were detected for Na-doped Cu_2_ZnSn(S,Se)_4_ films at different temperatures. This result also confirmed the formation of pure phase Na-doped Cu_2_ZnSn(S,Se)_4_. The illustration in Figure 2 presents the change in the peak location of the A_1_ mode. As shown in the inset, the A_1_ mode peaks slightly move to a lower wave value with the selenization temperature increasing, which may be ascribed to the replacement of some larger Se for S in the lattice with the increased temperature, leading to the expanding of the lattice constant, which is in agreement with the XRD result.

**Figure 3:**
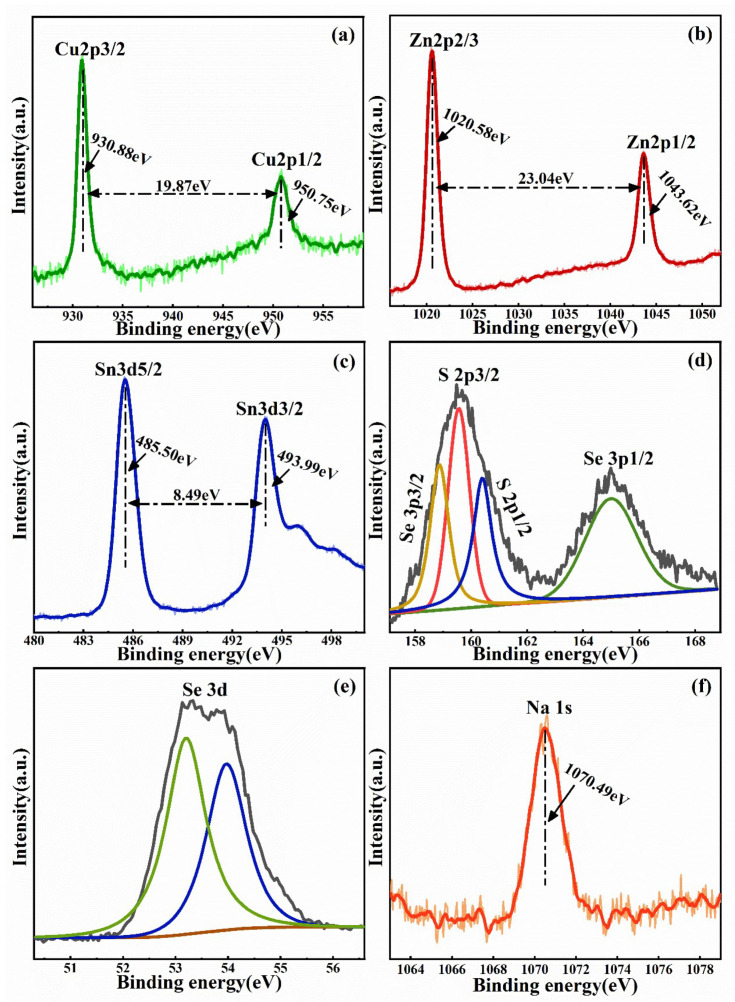
XPS spectra of Na-doped Cu_2_ZnSn(S,Se)_4_ film selenized at 540 °C: (**a**) Cu, (**b**) Zn, (**c**) Sn, (**d**) Se, (**e**) S and (**f**) Na.

The bonding states of the constituent chemical elements of the Na-doped Cu_2_ZnSn(S,Se)_4_ film selenized at 540 °C were studied by XPS measurements. As shown in Figure 3, the peaks corresponding to Cu 2p, Zn2p, Sn3d, S2p, Se3d, and Na1s were observed. Figure 3a presents the Cu 2p XPS spectra (green curve). The peaks at 950.75 and 930.88 eV accord with Cu 2p1/2 and Cu 2p3/2, and the interval value of the peaks is 19.87 eV. This indicates that Cu is present only in a Cu^+^ state [31]. Figure 3b reveals Zn 2p XPS spectra (red curve), two peaks situated at 1043.62 and 1020.58 eV were attributed to Zn 2p 1/2 and Zn 2p 3/2 peaks, respectively, and the binding energy interval is 23.04 eV, implying that Zn exists in the oxidation state of Zn^2+^ [32]. Figure 3c shows the XPS spectra of Sn 3d (blue curve), the peaks situated at 493.99 and 485.50 eV corresponding to Sn 3d 3/2 and Sn 3d 5/2, and the binding energy interval is 8.49 eV, indicating that Sn is presented in the oxidation state of Sn^4+^ [33]. The high-resolution spectra of the S 2p and the Se 3p peaks are displayed in Figure 3d. Due to the overlapping of the S 2p and the Se 3p core level, the XPS spectra were deconvoluted using a Gaussian fitting method. Four peaks lie at 158.86, 159.53, 160.38 and 165.02 eV, corresponding to the Se 3p 3/2, S 2p 3/2, S 2p 1/2, and Se 3p 1/2 peaks (yellow, red, blue and green curves), respectively. The peaks of S 2p 3/2 and S 2p 1/2 appeared at 159.53 and 160.38 eV, consistent with the standard reference value of S^2−^ [34]. As shown in Figure 3e, the XPS spectra of Se 3d were fitted by two peaks (green and blue curves) situated at 53.23 and 53.97 eV using a Gaussian fitting method. The two peaks are ascribed to Se 3d 3/2 and Se 3d 1/2, respectively, implying that the Se valence state is 2- [34]. Figure 3f presents the XPS spectrum (orange curve) of Na 1s with the peak at 1070.49 eV, suggesting the existence of monovalent Na^+^ in Na-doped Cu_2_ZnSn(S,Se)_4_ film [25]. The results of the XPS show that the Na-doped Cu_2_ZnSn(S,Se)_4_ film with the constituent element in the form of Cu^+^, Zn^2+^, Sn^4+^, S^2^^−^, Se^2^^−^ and Na^+^ has been successfully synthesized.

**Figure 4:**
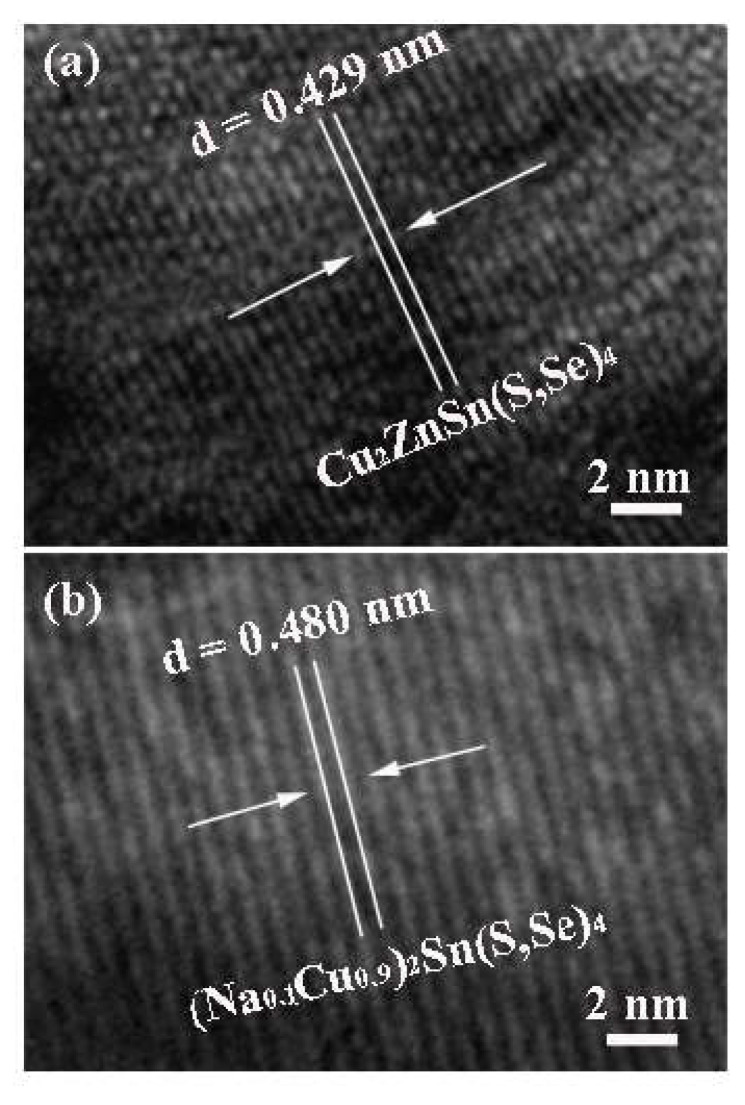
High-resolution TEM images of (**a**) Cu_2_ZnSn(S,Se)_4_ and (**b**) Na-doped Cu_2_ZnSn(S,Se)_4_ films selenized at 540 °C.

The TEM measurements were carried out to further clarify the specific microscopic structure of the Na-doped Cu_2_ZnSn(S,Se)_4_ film. Figure 4a,b display the TEM images of Cu_2_ZnSn(S,Se)_4_ and Na-doped Cu_2_ZnSn(S,Se)_4_ films selenized at 540 °C, respectively. The lattice streaks are distinctly observed in Figure 4a, and the lattice fringes with an interplanar spacing of 0.406 nm were put down to the (112) planes of kesterite CZTSSe [35]. As shown in Figure 4b, for the Na-doped Cu_2_ZnSn(S,Se)_4_ film, the fringe spacing evidently increased to 0.474 nm. CZTSSe interplanar spacing (0.406 nm) smaller than that (0.474 nm) of Na-doped Cu_2_ZnSn(S,Se)_4_ indicated an enlargement of the lattice due to the substitution of some Cu with Na atoms. According to the above results, it is inferred that Na replaced some Cu in the CZTSSe crystal lattice. TEM images indicate that we have obtained Na-doped Cu_2_ZnSn(S,Se)_4_ film with a highly uniform nanostructure, which is an important prerequisite for obtaining CZTSSe-based thin film solar cells with a higher efficiency.

It is well known that the chemical composition of the absorbing layer films influences device performance. Table 1 presents the EDS measurement results of the samples annealed at the different temperatures of 520, 540, and 560 °C (labeled as samples T520, T540, and T560, respectively). It was recognized that the atomic percentage of S evidently reduced from 8.47 to 2.08, and the atomic percentages of Se rose from 43.47 to 46.64 as the annealing temperatures varied from 520 °C to 560 °C. Hence, it was deduced that the atomic percentages of Se increased with the selenization temperatures increasing. Moreover, it can be acknowledged that the change in the atomic percentage for other elements (Cu, Zn, Na and Sn) was only slight in comparison to the variation in the contents of Se and S. The percentages of Se/(S+Se) were 83.69, 91.49, and 97.74 when the selenization temperatures are 520, 540 and 560 °C, respectively. This result directly provides evidence for the introduction of Se atoms into the lattice by high-temperature selenization, which verifies the conclusion that more Se was substituted for S as the selenization temperature increased in the above analysis results.

**Figure 5:**
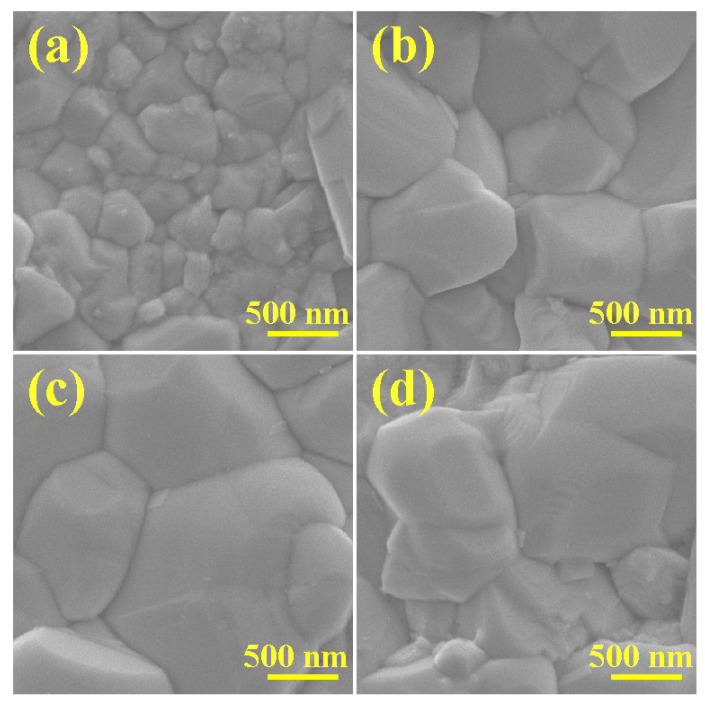
SEM images of Cu_2_ZnSn(S,Se)_4_ film selenized at (**a**) 540 °C and Na-doped Cu_2_ZnSn(S,Se)_4_ films selenized at (**b**) 520 °C; (**c**) 540 °C; (**d**) 560 °C.

The SEM surface images of the Cu_2_ZnSn(S,Se)_4_ selenized at 540 °C and the SEM images of the Na-doped Cu_2_ZnSn(S,Se)_4_ selenized at the different temperatures of 520, 540 and 560 °C displayed in Figure 5a–d. As displayed in Figure 5a, the surface morphology of the CZTSSe showed a relatively rough surface and irregular small grain of 500–800 nm. Figure 5b displays the surface morphology of the Na-doped Cu_2_ZnSn(S,Se)_4_ thin film selenized at 520 °C. Enhanced grain growth in the film was observed when Na was introduced, as the grain size was significantly enlarged to around 500 nm. When the selenization temperature increased to 540 °C, the evident morphological change of grain was observed. As displayed in Figure 5c, the crystalline size increased significantly to 1.0–2.5 μm, and the surface morphology was flat and dense. However, when the selenization temperature continually increased to 560 °C, the size of the crystal particle significantly decreased and the surface became rough, as displayed in Figure 5d. These results manifest that the optimum selenization temperature is 540 °C, which offers beneficial selenization surroundings to facilitate grain growth resulting in the formation of the compact thin film.

**Figure 6:**
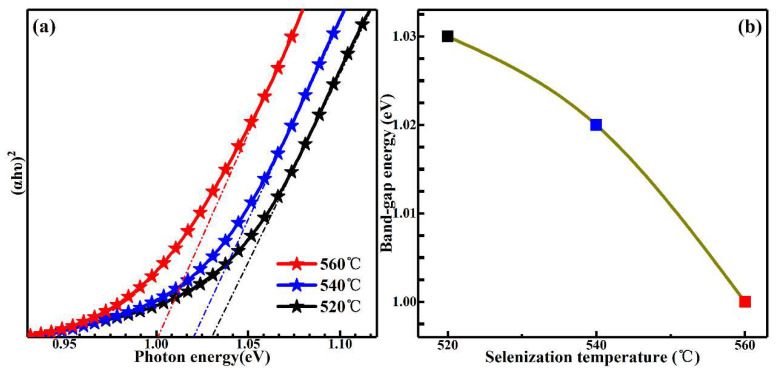
(**a**) The plot of (*αhυ*)^2^ vs. hυ for the absorption spectra; (**b**) band gap variation as a function of the selenization temperature.

To survey the impact of selenization temperatures on the optical band gaps of Na-doped Cu_2_ZnSn(S,Se)_4_, the optical absorption of the samples were obtained by a UV–VIS–NIR spectrophotometer. On the basis of the solid band theory, the relational expression between the energy of the incident ray (*hυ*) and the absorption coefficient (*α*) can be expressed as:

(*αhυ*) = *B*(*hυ* − *E*_g_)*^n^* (1)

where *h* is the Plank’s constant, *B* is the band edge constant, and *E*_g_ is the optical band gap. The value of *n* is 1/2, since CZTS is a direct band gap semiconductor [36]. The relationship of (*αhυ*)^2^ versus *hυ* for (Na_0.1_Cu_0.9_)_2_ZnSn(S,Se)_4_ has been shown in Figure 6a. Utilizing the data in Figure 6a and Equation (1), the band gaps are estimated to be 1.03, 1.02, and 1.00 eV for samples selenized at temperatures of 520, 540 and 560 °C, respectively. As shown in Figure 6b, we can clearly see that the band gaps show a declining trend. Combined with the XRD, Raman, TEM and EDS results, it is concluded that as the selenization temperature increased, the reduction in band gaps is ascribed to the change in lattice and difference in electronegativity due to alloying, as well as the modification of atomic structure owing to Se replacing the site of S. The adjustable band gap of Na-doped Cu_2_ZnSn(S,Se)_4_ film is of extraordinary importance for enhancing the performance of solar cells.

Table 2 lists the electrical properties of the films measured by Hall measurement. It is recognized that all Na-doped Cu_2_ZnSn(S,Se)_4_ films selenized at different temperatures show p-type conduction behaviors. When the selenization temperature increased, the carrier concentration first increased rapidly and then decreased slightly, and the carrier concentration reached a maximum value with 2.93 × 10^17^ cm^−3^ at the temperature of 540 °C. However, the resistivity first reduced rapidly and then rose slightly with the selenization temperature increasing, and reached a minimum value of 47.7 Ωcm at the temperature of 540 °C. Combined with the results of SEM, it was deduced that when the annealing temperature was increased from 520 to 540 °C, the crystal quality of the films was improved, the defects at the surface of films were passivated, and the resistivity and carrier concentration achieved the optimum value at the temperature of 540 °C. However, as the selenization temperature further increased from 540 to 560 °C, the surface morphology and crystallinity of the films began to deteriorate, leading to the resistivity and carrier concentration of the films worsening. By increasing the selenization temperature from 520 to 600 °C, the mobility decreased from 4.63 × 10^0^ cm^2^V^−1^S^−1^ to 4.63 × 10^−1^ cm^2^V^−1^S^−1^ and then increased to 2.57 × 10^0^ cm^2^V^−1^S^−1^. It is well known that conductivity is proportional to the product of mobility and carrier concentration, and for semiconductors, the behavior of a material can be very different depending on whether there are many carriers with low Hall mobility, or few electrons with high Hall mobility. In the present work, according to the Hall measurement results, it was observed that the change in mobility is indeed opposite to that of carrier concentration with the change in selenization temperatures. Finally, it was found that when the film was selenized at 540 °C, the electrical conductivity of Na-doped Cu_2_ZnSn(S,Se)_4_ film reached its optimal state with the lowest resistivity of 47.7 Ωcm, Hall mobility of 4.63 × 10^−1^cm^2^/Vs, carrier concentration of 2.93 × 10^17^ cm^−3^, and the film had higher carriers with low mobility. Thus, it implied that the selenization temperature of 540 °C may be more advantageous as this temperature supplied better annealing surroundings to form Na-doped Cu_2_ZnSn(S,Se)_4_ film.

**Figure 7:**
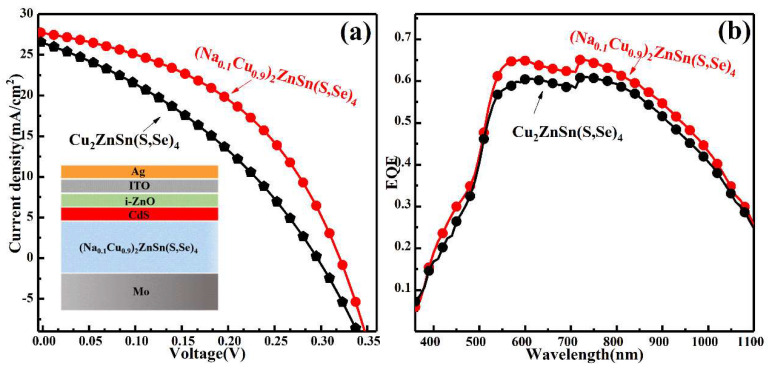
(**a**) J–V characteristics and (**b**) EQE of Cu_2_ZnSn(S,Se)_4_ and Na-doped Cu_2_ZnSn(S,Se)_4_ solar cells.

According to the above results, it was found that the Na-doped Cu_2_ZnSn(S,Se)_4_ film had better crystallinity and opto-electrical properties at the temperature of 540 °C, which are regarded suitable as the absorbed layer. Thus, it was regarded as the absorbed layer to fabricate a solar cell. Figure 7a displays the current density (J) against voltage (V) curves of the corresponding CZTSSe and (Na_0.1_Cu_0.9_)_2_ZnSn(S,Se)_4_ solar cells fabricated under the same experimental conditions. The illustration of Figure 7 shows a structural sketch of the device (SLG/Mo/(Na_0.1_Cu_0.9_)_2_ZnSn(S,Se)_4_/CdS/ZnO/ITO/Al) with a traditional cell structure. The detailed performance parameters of the two devices are presented in Table 3. The CZTSSe device exhibits a V_oc_ of 296 mV, a J_sc_ of 26.42 mA/cm^2^, an FF of 35.16%, and a PCE of 2.75%. However, the (Na_0.1_Cu_0.9_)_2_ZnSn(S,Se)_4_ device showed a PCE of 4.82% with V_oc_ of 338 mV, J_s_ of 27.16 mA/cm^2^, and FF of 52.59%. The V_oc_, J_sc_, and FF values of Na-doped Cu_2_ZnSn(S,Se)_4_ solar cells were enhanced compared with that of CZTSSe solar cells. According to the J–V curve graph, the shunt resistance (R_SH_) was evaluated to be ~130.80 Ω·cm^2^ and the series resistance (R_S_) was ~30.37 Ω·cm^2^ in the CZTSSe device, whereas R_SH_ is evaluated to be ~279.05 Ω·cm^2^ and R_S_ is ~17.58 Ω·cm^2^ for the Na-doped Cu_2_ZnSn(S,Se)_4_ solar cell. The increase in shunt resistance and decrease in series resistance is a consequence of the increase in crystal particle size and decrease in defect density in the Na-doped Cu_2_ZnSn(S,Se)_4_ device. These enhancements in shunt and series resistance led to the improvement of the current density and fill factor. The tendency of J_sc_, FF and V_oc_ resulted in the increase in PCE from ~2.75% to ~4.82%, suggesting that Na doping can enhance the performance of solar cells. To survey the spectral response and in-depth understanding of the performance improvement of the device’, the spectral responses EQE was measured for CZTSSe and (Na_0.1_Cu_0.9_)_2_ZnSn(S,Se)_4_ device, as displayed in Figure 7b. The EQE data were enhanced in the entire visible region for the Na-doped Cu_2_ZnSn(S,Se)_4_ device, which can be interpreted by the increased Rsh and reduced R_S,_ compared to the CZTSSe device. The results also revealed the preferable light carrier collection in Na-doped Cu_2_ZnSn(S,Se)_4_ solar cells, which was much enhanced in comparison with that of CZTSSe solar cells. Therefore, compared with the CZTSSe, the device obtained by using Na-doped Cu_2_ZnSn(S,Se)_4_ as the absorption layer had a better performance. Although there is still a discrepancy between the achieved efficiency and reported record efficiency, we will mainly explore and optimize the experimental condition of each part in the device structure to achieve the highly powered conversion efficiency for Na-doped Cu_2_ZnSn(S,Se)_4_ solar cells in our subsequent work.

## 4. Conclusions

The Na doping method has proven to be promising in the preparation of CZTS-based solar cells. The Na-doped Cu_2_ZnSn(S,Se)_4_ films were obtained at different selenization temperatures by the sol–gel method together with the rapid annealing technique. The crystallinity, electrical, and optical properties of Na-doped Cu_2_ZnSn(S,Se)_4_ films were highly influenced by the selenization temperatures. The annealing temperature of 540 °C supplied favorable selenization surroundings to promote the growth of the grain which brought about the formation of the compact thin film. The *E_g_* of Na-doped Cu_2_ZnSn(S,Se)_4_ films can be tuned from 1.03 to 1 eV by an increase in the annealing temperature from 520 to 560 °C. All Na-doped Cu_2_ZnSn(S,Se)_4_ thin films had p-type conduction characteristics, and the films with better electrical properties were obtained by adjusting the selenization temperature. In the end, the Na-doped Cu_2_ZnSn(S,Se)_4_ film possessing the preferable crystallinity as well as optical and electrical properties, was acquired at the optimal selenization temperature of 540 °C, which was regarded as the absorbed layer to prepare the solar cell, achieving a PCE of 4.82%. Therefore, the selenization temperature of 540 °C can be more advantageous to synthesize the Na-doped Cu_2_ZnSn(S,Se)_4_ solar cells because this temperature supplies favorable annealing surroundings to form a Na-doped Cu_2_ZnSn(S,Se)_4_ absorber.

## Figures and Tables

**Table 1:** EDS results of the Na-doped Cu_2_ZnSn(S,Se)_4_ films selenized at different temperatures ranging from 520 to 560 °C.

Sample	Cu (at%)	Zn (at%)	Sn (at%)	Na (at%)	S (at%)	Se (at%)	Se/(S+Se)
T520	19.57	16.12	9.18	3.19	8.47	43.47	83.69
T540	20.79	16.63	9.71	3.31	4.22	45.34	91.49
T560	20.56	16.63	10.21	3.88	2.08	46.64	97.74

**Table 2:** Electrical properties of the Na-doped Cu_2_ZnSn(S,Se)_4_ films selenized at different temperatures ranging from 520 to 560 ℃.

Sample	ρ (Ω·cm)	n (cm^−3^)	μ (cm^−2^V^−1^s^−1^)	Conduction Type
T520	5.59 × 10^1^	9.22 × 10^16^	4.63 × 10^0^	p
T540	4.77 × 10^1^	2.93 × 10^17^	4.63 × 10^−1^	p
T560	5.61 × 10^1^	5.54 × 10^16^	2.57 × 10^0^	p

**Table 3:** The parameters of CZTSSe and Na-doped Cu_2_ZnSn(S,Se)_4_ device performance.

Sample	V_oc_ (mV)	J_sc_ (mA/cm^2^)	R_s_ (Ωcm^2^)	R_sh_ (Ωcm^2^)	FF (%)	PCE (%)
CZTSSe	296	26.42	30.37	130.80	35.16	2.75
(Na_0.1_Cu_0.9_)_2_ZnSn(S,Se)_4_	338	27.16	17.58	279.05	52.59	4.82
